# Anticancer Effects of the Novel Pyrazolyl-Urea GeGe-3

**DOI:** 10.3390/ijms25105380

**Published:** 2024-05-15

**Authors:** Ashleigh Williams, Emma Cooper, Bethany Clark, Laura Perry, Marco Ponassi, Erika Iervasi, Chiara Brullo, Alexander Greenhough, Michael Ladomery

**Affiliations:** 1 Centre for Research in Biosciences, School of Applied Sciences, University of the West of England, Coldharbour Lane, Frenchay, Bristol BS16 1QY, UK; 2 Proteomics and Mass Spectrometry Unit, IRCCS Ospedale Policlinico San Martino, L.go. R. Benzi 10, 16132 Genova, Italy; 3 Department of Pharmacy, Medicinal Chemistry Section, University of Genova, Viale Benedetto XV, 3, 16132 Genova, Italy; chiara.brullo@unige.it

**Keywords:** pyrazole, novel anticancer drugs, cell proliferation, cell migration

## Abstract

In a screen of over 200 novel pyrazole compounds, ethyl 1-(2-hydroxypentyl)-5-(3-(3-(trifluoromethyl) phenyl)ureido)-1*H*-pyrazole-4-carboxylate (named GeGe-3) has emerged as a potential anticancer compound. GeGe-3 displays potent anti-angiogenic properties through the presumptive targeting of the protein kinase DMPK1 and the Ca2^+^-binding protein calreticulin. We further explored the anticancer potential of GeGe-3 on a range of established cancer cell lines, including PC3 (prostate adenocarcinoma), SKMEL-28 (cutaneous melanoma), SKOV-3 (ovarian adenocarcinoma), Hep-G2 (hepatocellular carcinoma), MDA-MB231, SKBR3, MCF7 (breast adenocarcinoma), A549 (lung carcinoma), and HeLa (cervix epithelioid carcinoma). At concentrations in the range of 10 μM, GeGe-3 significantly restricted cell proliferation and metabolism. GeGe-3 also reduced PC3 cell migration in a standard wound closure and trans-well assay. Together, these results confirm the anticancer potential of GeGe-3 and underline the need for more detailed pre-clinical investigations into its molecular targets and mechanisms of action.

## 1. Introduction

**Figure 1:**
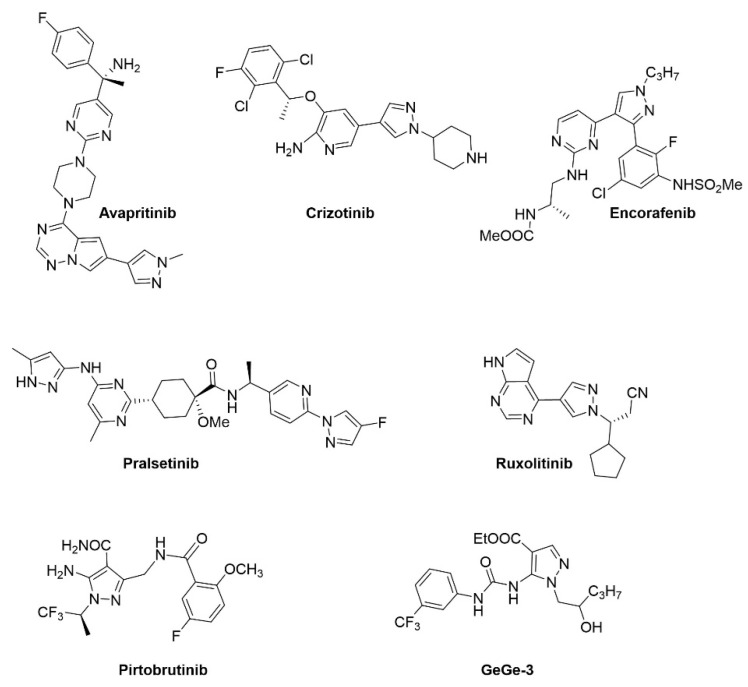
Molecular structure of a range of pyrazole-based drugs used in cancer therapy [9] compared to our lead compound ethyl 1-(2-hydroxypentyl)-5-(3-(3-(trifluoromethyl)phenyl)ureido)-1*H*-pyrazole-4-carboxylate (named GeGe-3).

Pyrazoles are molecules characterised by a 5-membered heterocycle with three carbon atoms and two adjacent nitrogen atoms. The pyrazole scaffold is very versatile and utilised in a wide range of drugs displaying a broad range of pharmacological activities, including anti-inflammatory, anticancer, anti-infective and many others [1,2,3]. One of the best-known examples of a successful pyrazole-based drug is the drug celecoxib, a nonsteroidal anti-inflammatory drug (NSAID) that selectively inhibits COX-2, used to treat pain and inflammation in a range of conditions [4]. There has also been a considerable amount of progress in developing pyrazole derivatives as potential anticancer drugs [5,6,7,8]. In the latter context, the pyrazole scaffold has proven to be especially useful for the design of protein kinase inhibitors. A significant proportion of novel, FDA (US Food and Drug Administration)-approved protein kinase inhibitors are based on pyrazole chemistry; these include avapritinib, crizotinib, encorafenib, pralsetinib, ruxolitinib, and pirtobrutinib [9]. Their structures are illustrated in Figure 1, compared to GeGe-3.

In fact, over the last ten years, more than 200 pyrazole compounds have been screened in the search for novel therapeutic agents for cancer treatment with an emphasis on identifying compounds that work through the modulation of angiogenesis. In this context, we have observed that several experimental pyrazole compounds were able to modulate ERK1/2, p38MAPK and AKT activities in VEGF-stimulated human umbilical vein endothelial cells (HUVECs). In particular, ethyl 1-(2-hydroxypentyl)-5-(3-(3-(trifluoromethyl) phenyl)ureido)-1*H*-pyrazole-4-carboxylate (henceforth named GeGe-3, Figure 1) emerged as a promising anti-angiogenic compound, with evidence suggesting that it targets dystrophia myotonica protein kinase 1 (DMPK1) [10]. GeGe-3 inhibits HUVEC (human umbilical vein endothelial cell) proliferation and endothelial tube formation, impairing inter-segmental angiogenesis during the development of zebrafish embryos and blocks angiogenesis in mice, and as a result impairs the growth of subcutaneously xenografted Lewis Lung Carcinoma cells [11]. Additional experiments, confirmed by in silico molecular docking studies, led to the identification of several additional candidate intracellular GeGe-3 targets that include calreticulin, a multifunctional Ca^2+^-binding protein [12].

Calreticulin displays multiple biochemical activities (including roles in calcium homeostasis, oxidative stress reactions and lectin binding). Because of these multiple biochemical activities, calreticulin is involved in distinct cellular processes including cell adhesion, cell–cell interactions, migration, phagocytosis, immune responses, cell proliferation, differentiation, and apoptosis [13,14,15,16]. Calreticulin dysfunction is also important in cancer, both in the context of tumor formation and at different clinical stages of the disease [17,18].

We originally demonstrated that GeGe-3 alters calcium intracellular traffic in HUVEC cells in a concentration dependent fashion, most likely due to its binding to calreticulin. This effect, in turn, alters the organization of the cytoskeleton, with F-actin moving to the cortical cell region, an expected result when calcium mobilization is altered. Focal adhesion kinase (FAK, also known as PTK2) expression is negatively modulated by the treatment of HUVEC cells with GeGe-3. For these reasons, it is likely that GeGe-3′s anti-angiogenetic activity is mediated by its binding to calreticulin, affecting calcium mobilization, F-actin localization and FAK down-regulation.

In subsequent studies, GeGe-3 was shown to affect crosstalk between pancreatic cancer cells and the tumor microenvironment. A major player in this crosstalk is annexin A1. It acts in a paracrine fashion to activate endothelial cells and is secreted through micro-vesicles. Its ability to interact with cell membrane phospholipids is also regulated by Ca^2+^. The annexin A1 mimetic peptide Ac2-26 can be used to promote endothelial cell motility; this process is blocked by GeGe-3, contributing to the inhibition of angiogenesis [19]. The inhibition of calcium mobilization in HUVEC cells caused by GeGe-3 also prevents the translocation of annexin A1 to the plasma membrane. The translocation of annexin A1 to the plasma membrane is dependent on phosphorylation of serine 27 by PKCa [20]. PKCa pathways appear strongly inhibited by GeGe-3, suggesting a mechanism for the failed localization of annexin A1 to the plasma membrane [19].

Taken together, these findings have prompted us to consider GeGe-3 as a priority compound in a library of potential antitumor agents developed by Dr Chiara Brullo and colleagues, requiring detailed investigation. The aim of the present study was to further explore the anticancer properties GeGe-3 by administering it to a wide range of cancer cell lines, measuring the effect on cell proliferation, viability, and migration.

## 2. Results and Discussion

### 2.1. Effect of GeGe-3 on Cell Proliferation

**Figure 2:**
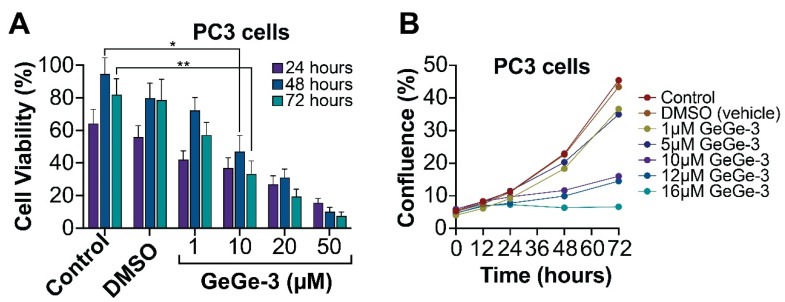
The effect of GeGe-3 on cell viability in PC3 prostate cancer cells. (**A**) Cells were seeded at 50,000 cells/mL and exposed to 1, 10, 20 and 50 μM GeGe-3. A trypan blue cell viability assay was performed at 24-, 48- and 72-h post-treatment. The percentage of viable cells is shown. *N* = 3. (**B**) Cell confluence was measured using the IncuCyte live cell imager over a 72-h period in PC3 cells. *N* = 6. Indicates significant differences (* *p* ≤ 0.05, ** *p* ≤ 0.01).

GeGe-3 is among a growing list of pyrazole-based compounds that display anticancer properties: several compounds have been tested for this purpose over the last decade and a half [21,22,23,24]. To investigate the effect of GeGe-3 on cell proliferation, we selected the well-studied prostate adenocarcinoma cell line PC3. Calreticulin, a GeGe-3 target, may affect prostate cancer progression; in PC3 cells, it promotes β1-integrin mRNA stability [25]. Integrins are receptors involved in cell adhesion with roles in metastasis, and targets for the development of therapies that prevent disease progression [26]. We seeded 50,000 PC3 cells per ml into 24-well plates and treated them with GeGe-3 at concentrations ranging from 1 μM to 50 μM. A viable cell count was performed at 24, 48 and 72-h after exposure to GeGe-3. We noticed a pronounced and progressive reduction in viable cell numbers over the 72-h interval, particularly at concentrations of 10 μM and above (Figure 2A). Next, we performed a cell confluence assay using an Incucyte instrument, focusing on a lower range of GeGe-3 concentrations (1–16 μM) as these concentrations were sufficient to exert an effect on PC-3 viable cell numbers. Cell confluence was measured over a 72-h period. Cells exposed to up to 5 μM GeGe-3 broadly increased their confluence comparable to the controls; when exposed to 10 μM and above, cell confluence did not increase significantly and even declined with 16 μM GeGe-3 (Figure 2B). Together, these experiments demonstrate that GeGe-3, at a concentration of 10 μM, severely restricts the ability of PC3 prostate cancer cells to proliferate.

### 2.2. Effect of GeGe-3 on Cell Metabolism

**Figure 3:**
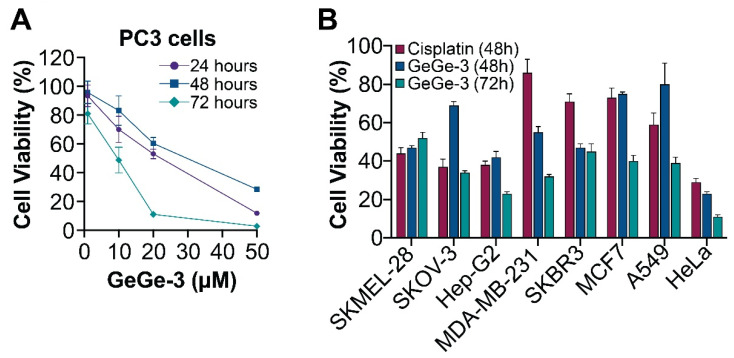
The effect of GeGe-3 on cell metabolism measured through the MTT assay. (**A**) A total of 4000 PC3 prostate cancer cells were seeded into 96-well plates and exposed to 1, 10, 20 and 50 μM GeGe-3 and an MTT assay performed at 24-, 48- and 72-h post-treatment; absorbance values are shown. *N* = 7 repeats in all experiments; *p* < 0.01. (**B**) GeGe-3 was tested at a fixed concentration of 10 μM against eight different cancer cell lines (namely, SKMEL-28 cutaneous melanoma, SKOV-3 ovarian adenocarcinoma, Hep-G2 hepatocellular carcinoma, MDA-MB231 breast adenocarcinoma, SKBR3 breast adenocarcinoma, MCF7 breast adenocarcinoma, A549 lung carcinoma, and HeLa cervical carcinoma using the chemotherapeutic drug cisplatin as reference compound (at a concentration of 10 μM). An MTT assay was performed at 48-h following both cisplatin or GeGe-3 treatment, and 72-h alone for GeGe-3 treatment. The data is shown as a percentage of the control (DMSO alone). *N* = 3 experiments.

The MTT assay is a widely used technique to measure cell viability through metabolism. The basis of the assay is that MTT, a yellow tetrazole, is actively reduced to purple formazan in living, metabolically active cells. We assessed the effect of GeGe-3 on cell metabolism, initially in PC3 cells, over a period of 72 h following exposure to GeGe-3. Control cells, or cells exposed to vehicle proliferated normally, but the addition of GeGe-3 (1, 10, 20 and 50 μM) appeared to decrease cell viability significantly. This increased over time and was most apparent 72 h after treatment (Figure 3A). 

To investigate the wider antiproliferative activity of GeGe-3, we then applied it at 10 μM concentration to a panel of cancer cell lines representing a range of solid tumors (Figure 3B). The results show that GeGe-3, at 10 μM, severely restricted the proliferation of all cancer cell lines tested. Liver cancer (Hep-G2), breast cancer (MDA-MB231), and cervical carcinoma (HeLa) appeared the most sensitive to GeGe-3, 48 h after exposure to 10 μM GeGe-3, and the values decreased further at 72 h, suggesting that the inhibitory effect of a single dose of GeGe-3 on cell proliferation may be relatively persistent. We also compared the effect of GeGe-3 to cisplatin, an established and widely used chemotherapeutic drug that interferes with DNA replication [27]. At 48 h, for MDA-MB-231 cells (breast cancer, invasive ductal carcinoma) the metabolic activity appeared to be more strongly affected by GeGe-3 compared to the reference compound cisplatin.

In summary, all the cell lines were adversely affected by GeGe-3. These results confirm the significant antiproliferative action of GeGe-3, further suggesting that there may be scope for its development as a chemotherapeutic drug. It will be imperative to investigate in further detail its molecular interactions and mechanism of action, which may differ from cancer to cancer due to the differential expression and activity of GeGe-3 targets in each cancer type.

### 2.3. Effect of GeGe-3 on Cell Migration and Invasion

**Figure 4:**
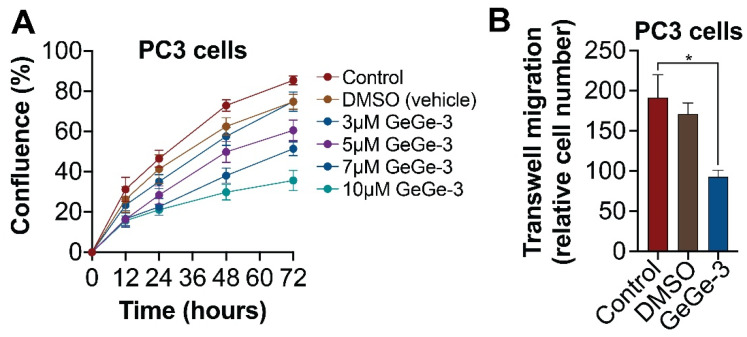
Effect of GeGe-3 on cell migration in PC3 cells. (**A**) IncuCyte Scratch Wound Analysis software (v2020C) was used to monitor the percentage wound closure of PC3 cells over a 72-h period following treatment with 3, 5, 7 and 10 μM GeGe-3; *N* = 8 replicates. (**B**) Trans-well cell migration assay following treatment with 20 and 50 μM GeGe-3. Relative cell numbers are shown. *N* = 3 repeats. * *p* < 0.05.

The emerging range of GeGe-3 targets include calreticulin [12], a multifunctional protein associated with many hallmarks of cancer, including several Ca^2+^-dependent processes associated, e.g., cell adhesion [14]. GeGe-3 has previously been shown to inhibit HUVEC cell migration [10]. We therefore decided to test the potential effect of GeGe-3 on cell migration in PC3 cells. We performed a scratch (wound closure) assay using an Incucyte system, testing the effect of a range of GeGe-3 concentrations (3, 5, 7 and 10 μM). These relatively lower concentrations of GeGe-3 significantly decreased the rate of wound closure (Figure 4A), with the 10 μM treatment resulting in only 44% wound closure. Original scratch assay images are shown in Supplementary Figure S1. To further assess cell migration, a trans-well assay was performed. The trans-well assay provided evidence that 20 µM of GeGe3 significantly decreased PC3 cell migration across a membrane (Figure 4B). The ability of GeGe-3 to inhibit cell migration further confirms the anticancer potential of the compound. More detailed preclinical investigations of GeGe-3, including detailed elucidation of its molecular targets and mechanisms of action, are now urgently needed.

## 3. Materials and Methods

### 3.1. Cell Culture and Reagents

The PC3 (prostate adenocarcinoma) cell line was sourced from ECACC. Other cell lines were: SKBR3, MCF-7 and MDA-MB321 (breast adenocarcinoma); SKMEL-28 (melanoma), HeLa (cervical adenocarcinoma) andHepG2 (hepatocellular carcinoma), sourced from the Biologic Bank and Cell Factory, IRCCS Policlinico San Martino, Genova, Italy. A549 (lung carcinoma) and SKOV-3 (ovarian adenocarcinoma) were sourced from ATCC, Manassas, VA, USA. Cells were maintained and cultured in Dulbecco’s Modified Eagles Medium (DMEM), and where required were supplemented by high glucose, GLUTAMAX (Thermofisher Scientific, Waltham, MA, USA). Growth media included 1% penicillin/streptomycin (Thermofisher Scientific) and 10% fetal bovine serum (FBS) (Sigma-Aldrich, St. Louis, MO, USA). Cells were grown in standard incubators at 37 °C and 5% CO_2_. GeGe-3 and cisplatin (the latter kindly provided by the pharmacy (UFA-Unità Farmaci Antiblastici) of the IRCCS Ospedale Policlinico San Martino in Genova) were dissolved in dimethyl sulfoxide (DMSO) (Sigma-Aldrich) to make a 10 mM stock solution and stored at −20 °C. GeGe-3 and cisplatin, after an intermediate dilution in culture medium, were added directly to cell culture media to achieve the desired concentrations.

### 3.2. Trypan Blue Cell Viability Assay

PC3 cells were plated into 24-well plates at a density of 50,000 cells/mL. Cells were then treated with a range of concentrations of GeGe-3 (1, 10, 20 or 50 µM). After 24-, 48- and 72-h, cells were trypsinized with TrypLE express (Thermofisher Scientific) and resuspended in DMEM. 10 µL of cell suspension was mixed with 40 µL of trypan blue dye (0.4% *w*/*v*) and a live cell count carried out in a hemocytometer (Neubauer chamber).

### 3.3. MTT Assay

The MTT (3-(4,5-dimethyl-2-thiazolyl)-2,5-diphenyl-2*H*-tetrazolium bromide) assay kit was purchased from Abcam. For the MTT assay on PC3 cells (Figure 3A), cells were seeded into 96-well plate at 4000 cells/well. Cells were pre-incubated for 72 h and then treated with varying concentrations of GeGe3 (1, 10, 20 or 50 µM). After the desired time (24, 48, 72 h), culture media was removed and replaced with 50 µL serum-free media Opti-MEM and 50 µL MTT reagent in each well and cells incubated for three hours. The MTT reagent was removed and 150 µL MTT solvent was added to each well and placed on an orbital shaker for 15 min. Absorbance was measured at 590 nm using a FLUOstar OPTIMA microplate reader. The MTT assay on the wider sample of cancer cell lines (Figure 3B) was performed as follows. After an intermediate dilution in growth medium, GeGe-3 and cisplatin were added to the cultured cells at a final working concentration of 10 μM and incubated for 48 or 72 h. At the end of the incubation, 30 μL of MTT at a concentration of 2 mg/mL in PBS, were added to each well and incubated for four hours. The supernatants were removed and 100 μL/well of DMSO were added to each well to dissolve the formazan precipitates and absorbances read after 20 min. Results are expressed as a percentage of the control samples (DMSO vehicle alone). The assay was repeated three times, and a single compound was tested six times. 

### 3.4. Cell Confluence Assay

PC3 cells were plated into 96-well plates at 5000 cells/well. Cells were left for 48 h to settle and then treated with increasing concentrations of GeGe-3 (3, 5, 7, 10 µM). The plates were then placed into the IncuCyte live cell imager (Sartorius) for 72 h to take hourly readings. Cell confluence over time was measured using the IncuCyte basic analyzer software (v2020C).

### 3.5. Scratch Assay

PC3 ells were plated in a 96-well Imagelock plate at 10,000 cells/well. Cells were then grown to confluence. Once confluent, cells were treated with mitomycin C (Sigma-Aldrich) at a 15 µM concentration for two hours to inhibit cell proliferation and mitotic events. After two hours, the IncuCyte Wound Maker 96-Tool was used to create a wound through each well. Media was then aspirated from the wells and wells washed with PBS. Wells were then treated with fresh media containing increasing concentrations of GeGe-3 (3, 5, 7, 10 µM). The plate was then placed into the IncuCyte for 72 h to monitor the rate of wound healing. The results were analysed using IncuCyte Scratch Wound Analysis software (v2020C).

### 3.6. Trans-Well Migration Assays

PC3 ells were seeded in a 6-well plate at 2,500,000 cells/well and incubated for 72 h. Cells were then treated with 20 µM GeGe3 or DMSO (vehicle) and incubated for a further 24 h. Cells were then trypsinised and counted using trypan blue exclusion dye. 24-well plates were prepared with 1 mL culture media. A Boyden chamber (purchased from Greiner Bio-one) was inserted and 500,000 treated cells in 500 µL serum-free media (Opti-MEM, Fisher Scientific, Hampton, NH, USA) were added to each chamber. Cells were then incubated for 48 h. Chamber media was removed, and the chamber was cleaned with a cotton swab to remove remaining non-migrated cells. Cells were then permeabilized in ice cold methanol for five minutes. Methanol was removed and chambers were placed in 25 µL/mL Hoechst stain (Sigma-Aldrich) for five minutes. Cells were then washed in PBS and chamber membrane insert was cut and fixed onto a glass slide with mounting gel. Slides were imaged with an Axio Observer inverted microscope.

### 3.7. Statistical Analysis of Data

Statistical testing was undertaken using GraphPad Prism 9 software. All graphical data is presented as mean ± standard error of mean (SEM). Trypan Blue, MTT, confluence and scratch assays were analysed using two-way ANOVA, followed by both Dunnett’s and Tukey’s post hoc testing to determine *p* values. Trans-well migration assay was analysed using one way ANOVA. Results were deemed significant if *p* values were ≤0.05 (*).

## 4. Conclusions

GeGe-3 is a novel pyrazolyl–urea compound that exhibits a distinctive anti-angiogenic potential in assays using HUVEC cells and zebrafish models and, as such, has emerged as a priority compound for detailed pre-clinical investigations to explore its anticancer potential. In this study we sought to test the compound on a range of well-established cancer cell lines. We find that at relatively low concentrations (10 μM) GeGe-3 clearly reduces cell counts and metabolic activity in PC3 prostate cancer cells. We further tested GeGe-3 on eight additional cell lines representing a range of solid cancers. GeGe-3 restricted cell growth at rates comparable to the established chemotherapeutic drug cisplatin. We also tested the effect of GeGe-3 on cell migration in PC3 cells and observed a noticeable reduction. Together, these results provide further evidence for the anticancer potential of GeGe-3 and consolidate its status as a priority compound for further preclinical evaluation.

## Data Availability

The data used and obtained during the study are available from the corresponding authors on request.

## Supplementary Materials

The following supporting information can be downloaded at: https://www.mdpi.com/article/10.3390/ijms25105380/s1.
