# Supplementary figures and images for: Anticancer Effects of the Novel Pyrazolyl-Urea GeGe-3
Source: Int J Mol Sci. 2024 May 15;25(10):5380. doi: 10.3390/ijms25105380 (PMC11121338; doi:10.3390/ijms25105380)

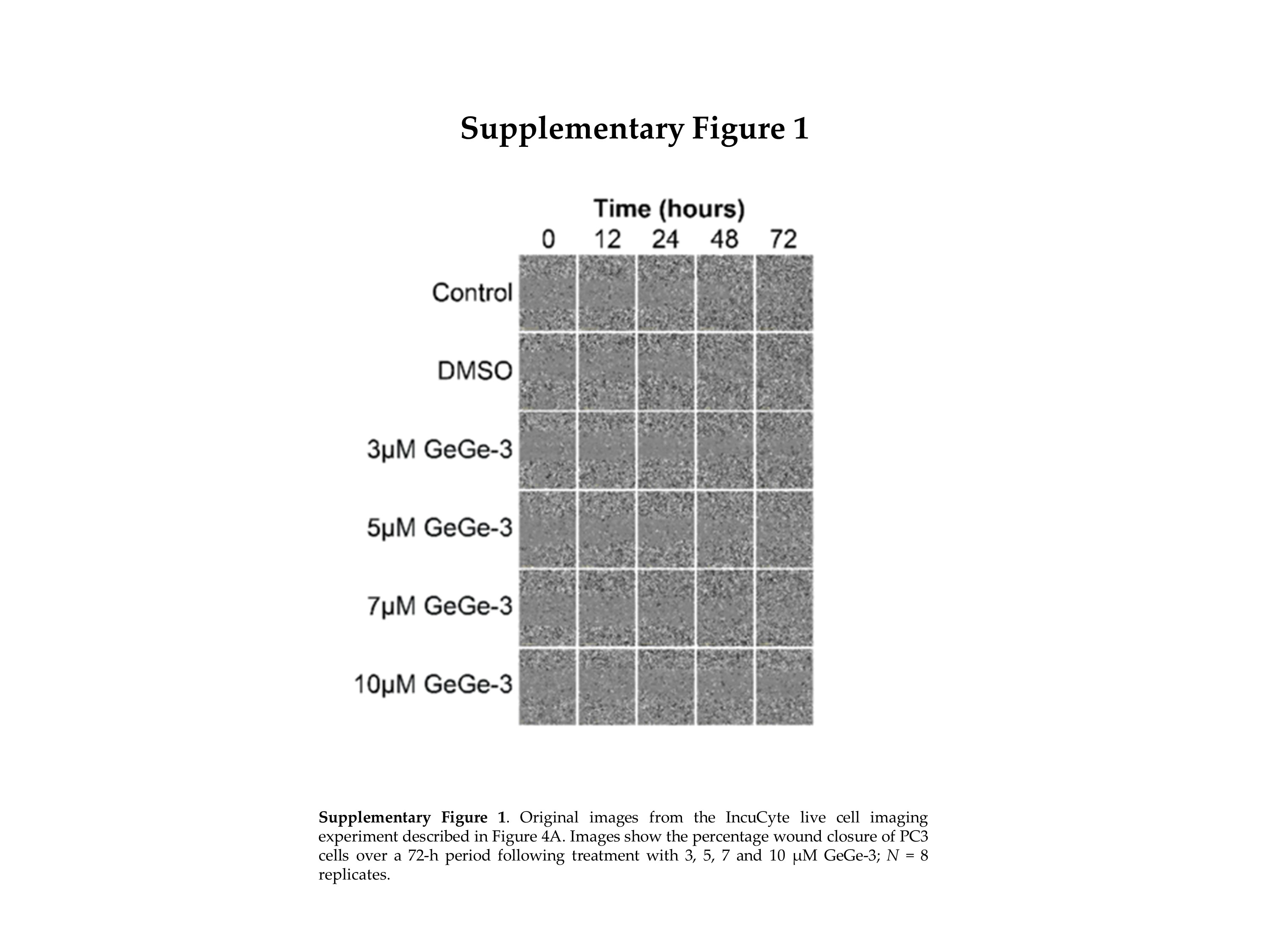

Supplement: Supplementary file 1 [file ijms-25-05380-s001.zip › ijms-2951787-supplementary.jpg]
